# Supplementary material for: Discrete and continuous mechanisms of temporal selection in rapid visual streams
Source: Nat Commun. 2017 Dec 5;8:1955. doi: 10.1038/s41467-017-02079-x (PMC5717232; doi:10.1038/s41467-017-02079-x)
Supplement: Supplementary file 1 — Supplementary Information [file 41467_2017_2079_MOESM1_ESM.pdf]

Supplementary information

Supplementary figures

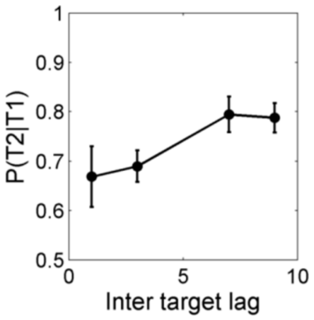

Supplementary Figure #1. Proportion of correct T2 identification (± s.e.m.) as a function of inter-target lag. Correct trials were defined as trials where subjects identified T2 in one of their guesses.

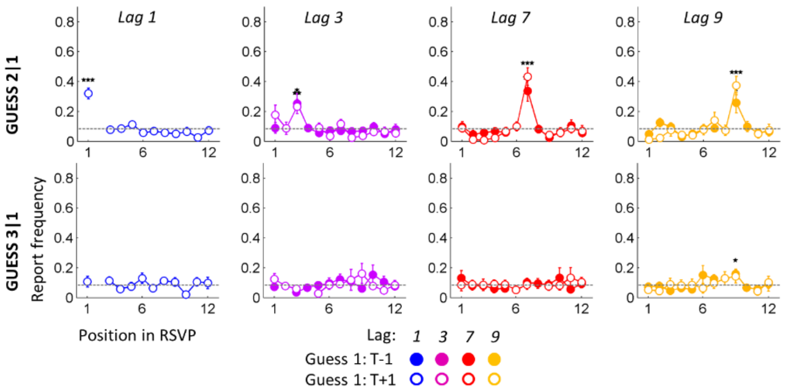

Supplementary Figure #2. Effect of Guess 1 position on the distributions of Guess 2 and 3. Rows represent Guess 2 and 3 and columns represent inter-target lags (blue, magenta, red and orange for Lag 1, 3, 7 and 9 respectively). Trials were split according to Guess 1 position: Closed symbols: subjects reported T-1 as Guess 1; Open symbols: subjects reported T+1 as Guess 1. For each position in the RSVP, the proportion of trials was compared to the chance level. A significant difference is indicated by an asterisk (*: P < .05; **: P < .01; ***: P < .001, error bars represent standard error to the mean).

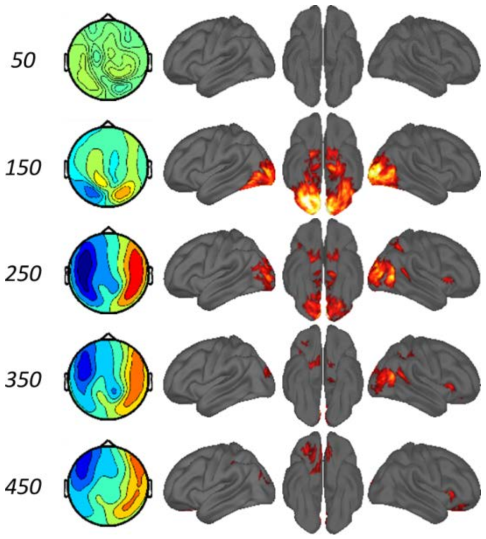

Supplementary Figure #3. Example of evoked activity during the localizer task. Topographies represent magnetic fields evoked by the presentation of face stimuli. The corresponding sources are represented on the right (left, inferior and right view of the brain).

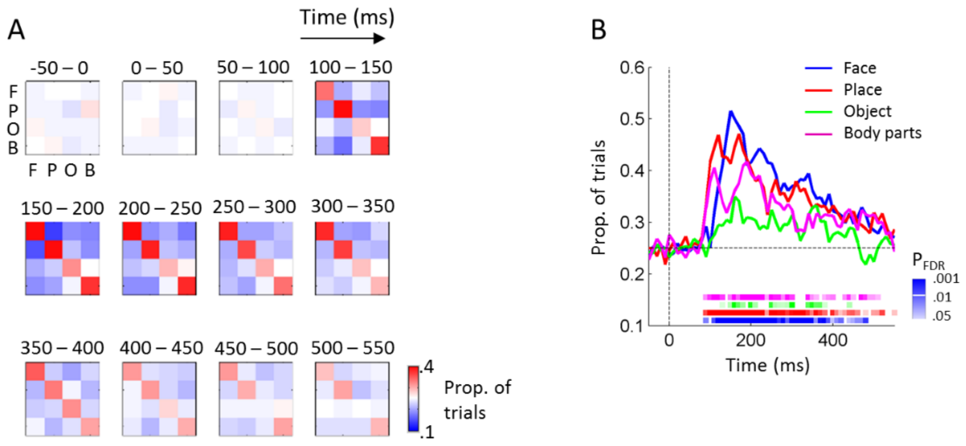

Supplementary Figure #4. Classifiers' confusion matrices. (A) Confusion matrices as a function of time for category classifiers (F: Face, P: Place, O: Object, B: Body parts). Colors represent the proportion of trials. (B) Time courses representing the diagonal of the confusion matrices for each stimulus category. Thick lines below the X axis represent significant signed rank tests for each category (FDR corrected across time and categories) with darker colors representing lower P-values.

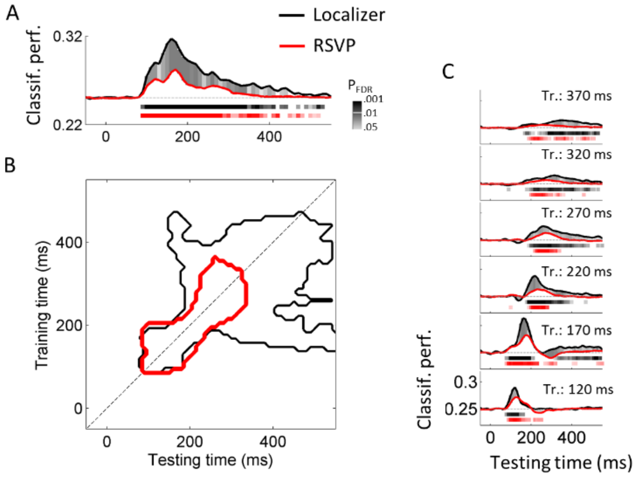

Supplementary Figure #5. Dynamics of brain activity in the Localizer (black) and in the RSVP (red) tasks. (A) Classification performance as a function of time in the two tasks. Classifiers were trained and tested on the same time sample. (B) Contour plot representing the thresholded temporal generalization matrices for the two tasks (threshold: .255). (C) Time courses of classifiers trained at specific times from 120 to 370 ms. Results from signed rank tests comparing classification performance to chance are represented by the thick line below the X axis. Signed rank tests comparing task conditions are represented by areas filled with grey, darker colors representing lower P-values. FDR correction for multiple comparisons was applied across specific training times (120 to 370 ms), testing times and conditions (RSVP and localizer).

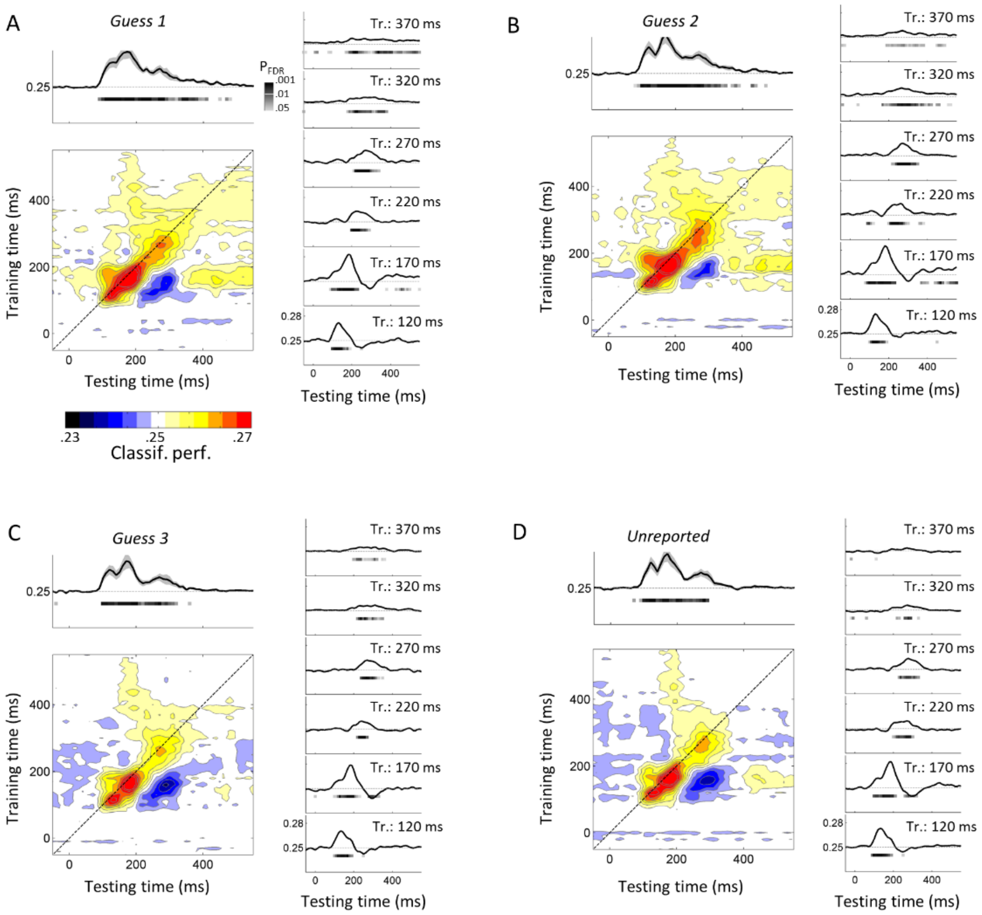

Supplementary Figure #6. Time-resolved decoding of reported stimuli. Panels represent classification performance for stimuli reported as Guess 1, Guess 2, Guess 3 or unreported stimuli (A, B, C and D respectively). In each panel: Top graph, one classifier was trained at each time sample and tested on the same time sample. The shaded grey area represents the standard error to the mean across subjects. The thick line below the X axis marks time samples at which the classification performance was significantly different from chance (Signed rank tests, $P_{FDR} < .05$). Right graphs: Classifiers were trained at specific time samples (from 120 to 370 ms) and tested on all other time samples. Matrix plot: Classifiers were systematically trained on each time sample and tested on all others. The color code represents the classification performance and the dotted line the diagonal of the matrix. Results from signed rank tests comparing classification performance to chance are represented by the thick line below the X axis with darker colors representing lower P-values. FDR correction for multiple comparisons was applied across specific training times (120 to 370 ms), all testing times and conditions (Guess 1, 2, 3 and unreported).
